# Characterization of VP1 sequence of Coxsackievirus A16 isolates by Bayesian evolutionary method

**DOI:** 10.1186/s12985-016-0578-3

**Published:** 2016-07-28

**Authors:** Guolian Zhao, Xun Zhang, Changmin Wang, Guoqing Wang, Fan Li

**Affiliations:** Department of Pathogenobiology, The Key Laboratory of Zoonosis, Chinese Ministry of Education, Norman Bethune College of Basic Medicine, Jilin University, Changchun, Jilin 130021 China

**Keywords:** Coxsackievirus A16, HFMD, Molecular evolution, Genetic diversity, Bayesian method

## Abstract

**Background:**

Coxsackievirus A16 (CV-A16), a major etiopathologic cause of pediatric hand, foot, and mouth disease (HFMD) worldwide, has been reported to have caused several fatalities. Revealing the evolutionary and epidemiologic dynamics of CV-A16 across time and space is central to understanding its outbreak potential.

**Methods:**

In this study, we isolated six CV-A16 strains in China’s Jilin province and construct a maximum clade credibility (MCC) tree for CV-A16 VP1 gene by the Bayesian Markov Chain Monte Carlo method using 708 strains from GenBank with epidemiological information. The evolution characteristics of CV-A16 VP1 gene was also analysed dynamicly through Bayesian skyline plot.

**Results:**

All CV-A16 strains identified could be classified into five major genogroups, denoted by GI–GV. GIV and GV have co-circulated in China since 2007, and the CV-A16 epidemic strain isolated in the Jilin province, China, can be classified as GIV-3. The CV-A16 genogroups circulating recently in China have the same ancestor since 2007. The genetic diversity of the CV-A16 VP1 gene shows a continuous increase since the mid-1990s, with sharp increases in genetic diversity in 1997 and 2007 and reached peak in 2007. Very low genetic diversity existed after 2010. The CV-A16 VP1 gene evolutionary rate was 6.656E-3 substitutions per site per year.

**Conclusions:**

We predicted the dynamic phylogenetic trends, which indicate outbreak trends of CV-A16, and provide theoretical foundations for clinical prevention and treatment of HFMD which caused by a CV-A16.

**Electronic supplementary material:**

The online version of this article (doi:10.1186/s12985-016-0578-3) contains supplementary material, which is available to authorized users.

## Background

In the pediatric population, hand, foot, and mouth disease (HFMD) is a common self-limiting condition caused by various serotypes of the enterovirus A species that is typically characterized by fever, pharyngalgia, malaise, erythema and herpetic lesions on hands and feet, as well as exanthema on oral mucosa and tongue [1]. A minority of patients develop severe neurologic complications such as acute flaccid paralysis, encephalitis, pulmonary edema, and myocarditis, with fatal outcomes in some severely afflicted patients [2, 3]. Over the last few decades, HFMD has been a very common pediatric infection in the Asia–Pacific region, with sporadic outbreaks reported in Europe and North America. In mainland China, HFMD has been listed as a notifiable disease since 2008 [4–6].

Coxsackievirus A16 (CV-A16) and enterovirus 71 (EV-A71) were major pathogens of HFMD in the past few decades, with EV-A71 more frequently associated with neurologic diseases [7]. Vaccine trials for the first EV-A71 vaccine have reached phase 3 clinical testing in China, with an estimated vaccine protection rate of 90 % against clinical EV-A71 infection-associated HFMD and 80.4 % against other EV-A71-associated diseases [3]. The other major pathogen of HFMD, CV-A16 has had epidemic presence in China and abroad for many years, but generating much lower social concern compared to EV71.

The first CV-A16 was identified in 1951 in South Africa, although there were no reports of an HFMD epidemic at the time [8]. New Zealand reported the first case of HFMD in the world in 1957 [9]. The relationship between CV-A16 and HFMD was confirmed by Atsop in 1959, and officially coined the term “Hand, Foot and Mouth Disease” based on clinical symptoms [10]. Although it is the first-identified HFMD virus, there is no specific antiviral treatment for CV-A16, which thus deserves more research attention. Recent years have seen extensive research efforts toward phylogenetic analysis of the HFMD pathogen based on bioinformatics in order to understand correlations between virus genogroup changes and disease epidemic trends [8, 11, 12]. At present, most extant phylogenetic trees are constructed based on neighbor-joining (NJ) distance, maximum parsimony (MP), and maximum likelihood (ML) [8, 13]. In reality, however, the difference between sequences does not completely represent the evolutionary distance, with large potential for errors [14]. The posterior probability is derived based on the Markov chain by Bayesian statistics, which allow researchers to use prior knowledge for guiding the construction of phylogenetic trees and to infer the maximum posteriori probability for estimating the most likely phylogenetic tree [15]. Moreover, the Bayesian method uses the posterior probability to visually represent phylogenetic relationships, thereby eliminating the need for bootstrapping [15, 16], and is widely used to construct the phylogenetic trees of swine-origin influenza A (H1N1) virus, measles viruses (MV), and EV-A71 and for accurate judgments of the relationship between genetic diversity (g) and epidemics of associated diseases [16–18].

This study was undertaken to investigate evolutionary and epidemiological dynamics of HFMD, with particular focus on CV-A16 genetic history and dynamics, within and between countries where this disease is endemic to facilitate prediction of its emergence in new locations as well as to provide the basis for an effective public health response framework. Bayesian analyses were performed to construct maximum clade credibility (MCC) tree for all sequenced and downloaded sequences, and global population dynamics of CV-A16 over the previous 30 years were reconstructed to examine temporal trends in genetic diversity and association with major epidemics.

## Methods

### Sample collection

A total of 52 specimens, as well as information on patient demographics, clinical symptoms, and complications were obtained from 2012 to 2013 at Disease Control and Prevention Center of Changchun, China. Stool specimens were processed as described previously for subsequent RNA extraction [19]. Specimens of other types were used directly for viral RNA extraction. The strains used for sequencing were amplified and isolated in RD (rhabdomyosarcoma) cells as previously described [20].

### RNA extraction, RT-PCR, and sequencing

Viral RNA was extracted using QIAamp Viral RNA MiniKit (QIAGEN, USA). TaKaRa RNA PCRTM Kit (AMV) was used to do the amplification fragment according to previous reports. The real-time PCR (RT-PCR) was firstly performed to detect the presence of the common (universal) sequence of enterovirus (EV-F, EV-R), and the specific sequences of EV-A71 (EV71S-F, EV71S-R) and CV-A16 (CV-A16S-F, CV-A16S-R). Then using primers (CV-A16VP1-F and CV-A16VP1-R) amplified CV-A16 VP1 whole sequence. Primers and reaction conditions was shown in Additional file 1: Table S1. All results of RT-PCR products were analyzed by 1 % agarose gel electrophoresis [21]. A part of CV-A16 VP1 sequences have been submitted to GenBank (Accession no. KT000389-KT000394).

### Sequence collection and phylogenetic analyses

For phylogenetic analysis, a total of 759 CV-A16 VP1 gene sequences before June 2013 were downloaded from GenBank. We retrieved 706 sequences which known collection dates and isolate country for analysis. The accession numbers and specific information of the sequences was listed in Additional file 2: Table S2. These nucleotide sequences were isolated mainly from large HFMD outbreaks and sporadic cases that occurred globally over 1981–2013. Combined with the six sequences isolated from our laboratory, a total of 708 sequences were used in phylogenetic analysis.

### Alignment processing and recombination detection

The complete VP1 sequence alignment of the CV-A16 strains was conducted with the Clustal W program in MEGA 6.0. Excess sequence was cut off, and FASTA format that can be used in the BEAST 1.8.2 was exported. Then we use the SEAL (sequence simulation and alignment evaluation software, http://tree.bio.ed.ac.uk/software/seal/) software to edit the nucleotide sequence. RDP3 Restructuring Package was used to detect the recombination of all CV-A16 sequence [22]. Then DAMBE was used for the saturation monitoring, if ISS < ISS.c and *p* = 0.0000 (extremely significant), then these sequences were unsaturated and suitable for construction of phylogenetic tree [23]. Finally we calculated the best alternative model With JModeltest [24]. The calculation was done after the selection of all the four kinds of patterns and then the statistical of AIC value. The smaller of the AIC value, the better fitting for the model with the data. Then, usually we choose the model with smallest AIC value for the construction of phylogenetic trees.

### Bayesian Markov Chain Monte Carlo evolutionary analysis

Bayesian Markov chain Monte Carlo (MCMC) methods were used to construct a maximum clade credibility tree (MCC) using BEASTv1.8.2 (http://beast-mcmc.googlecode.com/files/BEASTv1.8.2.tgz). Tracer v1.6 (http://beast.bio.ed.ac.uk/Tracer) was used to output analysis of sampling data, and then the Tree Annotator program was employed to output the results of MCC tree model. In the end the MCC molecular evolutionary tree graph was illustrated with FigTree1.3 (http://tree.bio.ed.ac.uk/). At the same time, Bayesian skyline plot analyses was used to reconstruct the population history of CV-A16 by measuring the dynamics of VP1 gene genetic diversity over time with 160 typical CV-A16 VP1 (Additional file 3: Figure S1).

JModeltest result revealed that HKY was the best substitution model, and the molecular clock model chosen the Relaxed Clock: Uncorrelated Log-normal. Using the Bayesian Markov Chain Monte Carlo framework, 80 million steps were run, sampling every 8000 and removing 10 % as burn-in. Convergence was assessed using Tracer (v1.6), and effective sample size (ESS) values above 200 were accepted.

## Results

### Phylogenetic analysis of CV-A16

**Fig. 1:**
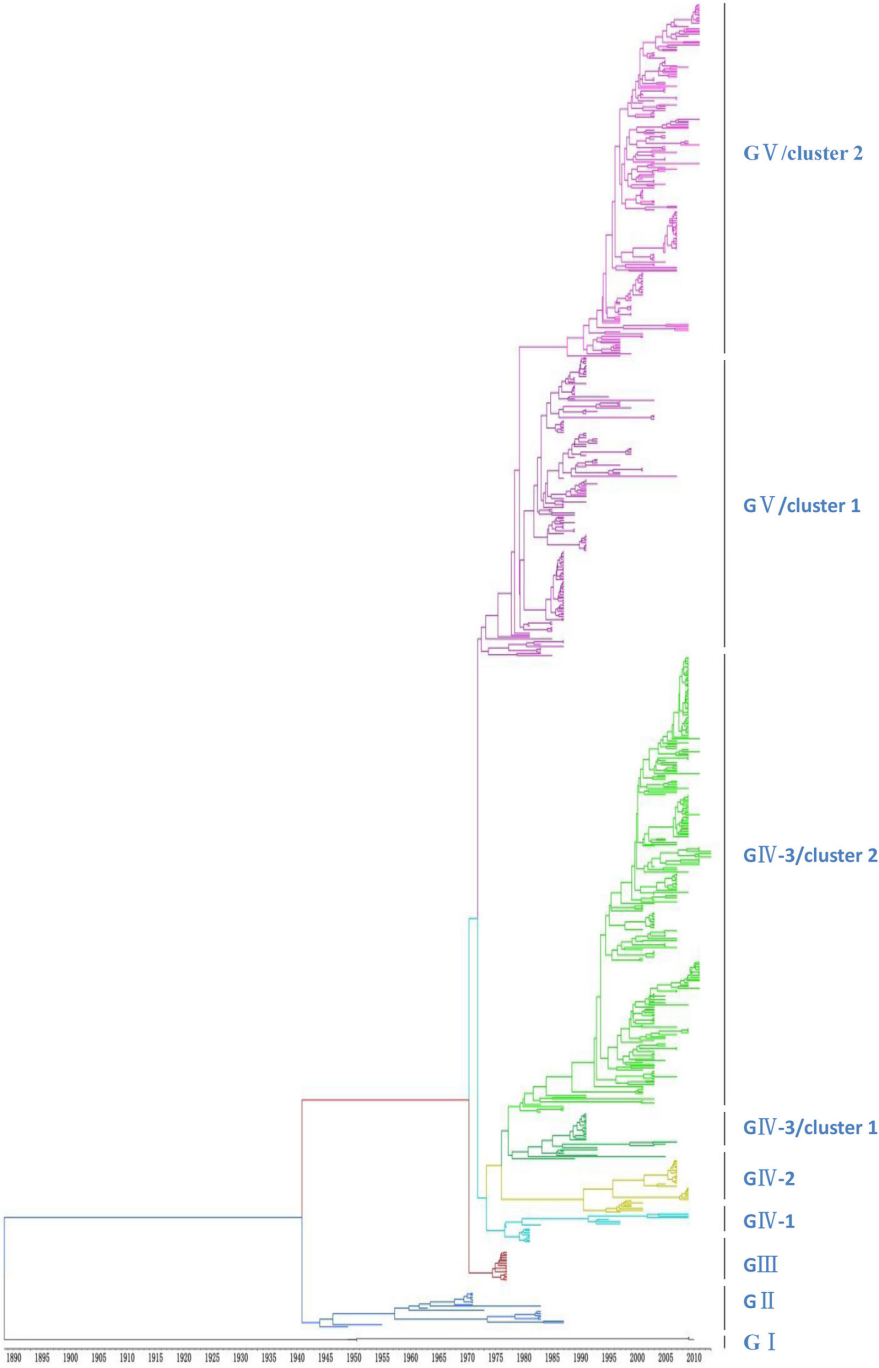
The maximum clade credibility (MCC) tree was estimated by Bayesian analysis of 708 CV-A16 complete VP1 sequences between 1951 and 2013. The phylogeny include 6 sequences from our laboratory as well as 702 sequences from GenBank with known collection dates and isolate country. Branches arecolor-coded according to the different genogroups. All sequences were classified into five genogroups, denoted GI–GV, Further, genogroup GIV could be divided into sub-genogroups GIV-1, GIV-2, and GIV-3. For the year and isolate country for each sequence, see Additional file 4: Figure S2 in the supplemental material

A total of 708 sequences (including six complete VP1 genome sequences determined by our laboratory) with complete VP1 region of CV-A16 were included in the phylogenetic analysis. Samples collected from 14 discrete locations in Southeast Asia (China, Japan, Malaysia, Russia, Australia, Cameroon, South Africa, France, Saudi Arabia, South Korea, Sweden, Taiwan, Thailand, and Viet Nam) between 1951 and 2013. Phylogenetic analyses were performed with Bayesian Markov chain Monte Carlo (MCMC) method. All CV-A16 strains identified could be classified into five major genogroups, denoted by GI–GV (Fig. 1). Further, the genogroup GIV could be divided into sub-genogroups GIV-1, GIV-2, and GIV-3. The genogroup GI, which no longer played a dominating role in contemporary epidemics, was represented by a prototype isolate (G-10), isolated in South Africa approximately 60 years earlier. Following its initial isolation, the genogroup GI was detected in China in 2008 and 2010 (EU812514 and JQ315094). Phylogenetic analysis revealed that genogroup GII was first reported from Japan in 1981 (AB465366). Among the 19 GII grouptypes, 15 were isolated from Japan during 1981–1998, and the other four from Malaysia during 1998–2000, with no further no genogoups reported thereafter. Interestingly, CV-A16 strains isolated in 1995 in Yamagata, Japan, were uniquely grouped into genogroup GIII, except for Y95-2260 (AB634302). After 1995, genogroups GIV and GV showed widespread co-circulation in many countries; 16 GIV-1 sub-grouptypes were composed of strains in Yamagata in Japan (1997), Taiwan (1998 and 2005), and in Russia (2004). Following 2005, this genigroup emerged in Wuhan, China, until 2011. Whereas 29 strains from Malaysia in 2005–2007, Russia in 2009–2010, France in 2010, and Japan (Yamagata) in 2011 were composed of GIV-2. From 1998 to 2013, the GIV-3 was distributed across eight countries, including Australia, Saudi Arabia, Japan, Malaysia, Russia, Cameroon, Taiwan, and China. The genogroup GV (353/708) originated from Malaysia in 1997, where the epidemic lasted 10 years (Table 1). Around 2007, the widely prevalent genogroups (GIV-3 cluster 1 and GV cluster 1) that caused major HFMD epidemics after 1995 appeared all to have been replaced by the new cluster (cluster 2), which has since dominated and circulated endemically in the Asia–Pacific region, generating major CV-A16-associated HFMD outbreaks (Fig. 1).

**Table 1:** Global distribution of genogroups of CV-A16

Isolated time and location	MCC	NJ
1951 South Africa	GI	A
2008 China: Fuyang		
2010 China: Ningbo		
1981–1998 Japan	GII	B2
1998–2000 Malaysia		
1995 Japan: Yamagata	GIII	B1a
1995, 1997 Japan: Yamagata	GIV-1	B1a
1998, 2005 Taiwan		
2004 Russia		
2011China: Wuhan		
2005–2007 Malaysia	GIV-2	B1c
2009–2010 Russia		
2010 France		
2011 Japan: Yamagata		
2000–2001 Australia	GIV-3 cluster1	B1b
2000 Malaysia		
2001, 2003 Arabia;		
2000, 2002–2003 Japan: Toyama		
2007–2008 China		
2007 Malaysia	GIV-3 cluster2	B1b
2009 Cameroon		
2007–2010 Russia		
2011 Japan: Yamagata		
2007–2013 China		
1997–2003, 2005–2007 Malaysia	GV cluster1	B1a
1998 Sweden		
2000–2004, 2006, 2008 Japan: Yamagata		
1999 Australia		
2000–2002 Thailand		
2008 South Korea		
2002 Japan Toyama		
2010 France		
2005 Australia	GV cluster2	B1a
2006 Taiwan		
2005, 2007 Malaysia		
2005 Thailand		
2008–2010 Japan: Yamagata		
2007 Japan: Toyama		
2005 Vietnam		
2010 Thailand		
2007–2012 China		

Note: The time and spatial distribution of all genogroups were shown in this table. The comparison the genogroups is constructed by maximum likelihood

### Origin and distribution of CV-A16 from Japan, Malaysia, and China

**Fig. 2:**
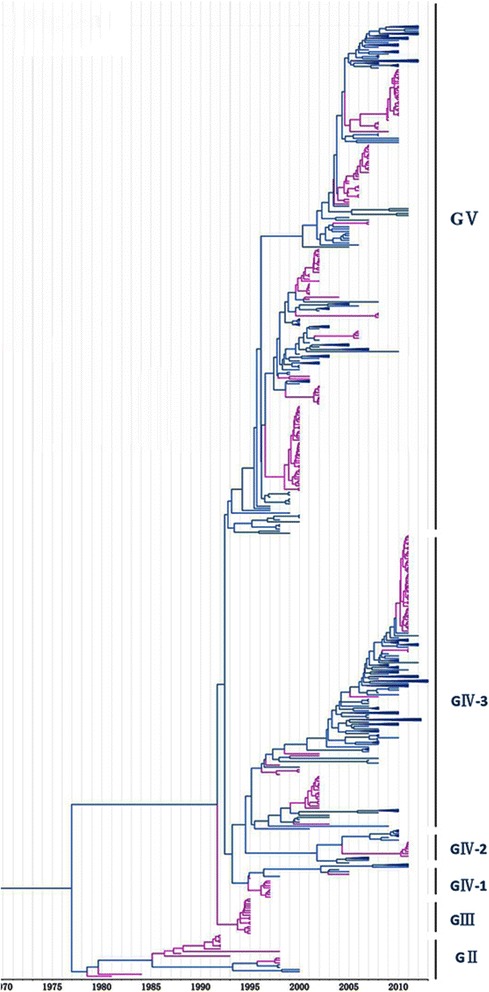
Phylogenetic analysis of CV-A16 from Japan. The scale is in units of evolutionary time in years. Phylogenetic tree of 255 Japan strains from 1981 to 2010. The branches of sequences from Japan are highlighted in purple

We, furthermore, studied country-specific epidemic strains to evaluate the trend of CV-A16 epidemic genogroups associated with serious HFMD outbreaks in Japan, Malaysia, and China. From the data sets shown in Fig. 2, we collected 255 CV-A16 strains from Japan and identified that the CV-A16 causing HFMD originated in Toyama in 1981. Subsequently, across 12 years, all CV-A16 epidemic strains belonged to genogroup GII in Japan; thereafter, genogroup GIII became the new epidemic strain until 1995. Shortly thereafter, genogroups GIV and GV replaced GIII and became the predominant genogroups since 1997. The epidemic strains GIV and GV circulated in 1997–2002 (85/255) and 2000–2010 (139/255), respectively. Interestingly, the Japanese epidemic strains (e.g., 1779-Yamagata-2011) in 2011 showed a close relationship to strains isolated during 2010–2012 from China (Figs. 2 and 5), as they all belonged to the GV genogroup, suggesting that the China epidemic strain invaded Japan in 2011.

**Fig. 3:**
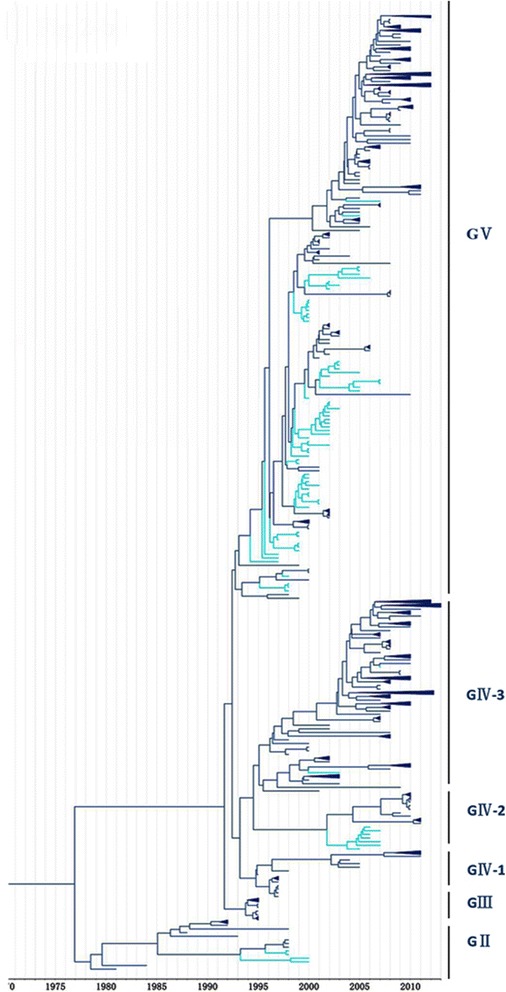
Phylogenetic analysis of CV-A16 from Malaysia. The scale is in units of evolutionary time in years. Phylogenetic tree of 81 Malaysia strains from 1998 to 2007. The branches of sequences from China are highlighted in blue

In our study, 81 CV-A16 epidemic strains from Malaysia were collected, of which genogroup GV was the most dominant, accounting for 83 % (68/82) (Fig. 3). Four strains belonging to genogroup GII were detected in Malaysia only in 1998–2000. The GIV genogroup (9/82) was a minor epidemic strain discovered in Malaysia in 2006–2007 and closely resembles the strains (Additional file 3: Figure S1, Fig. 5) detected in France, Japan, and Russia around 2010.

**Fig. 4:**
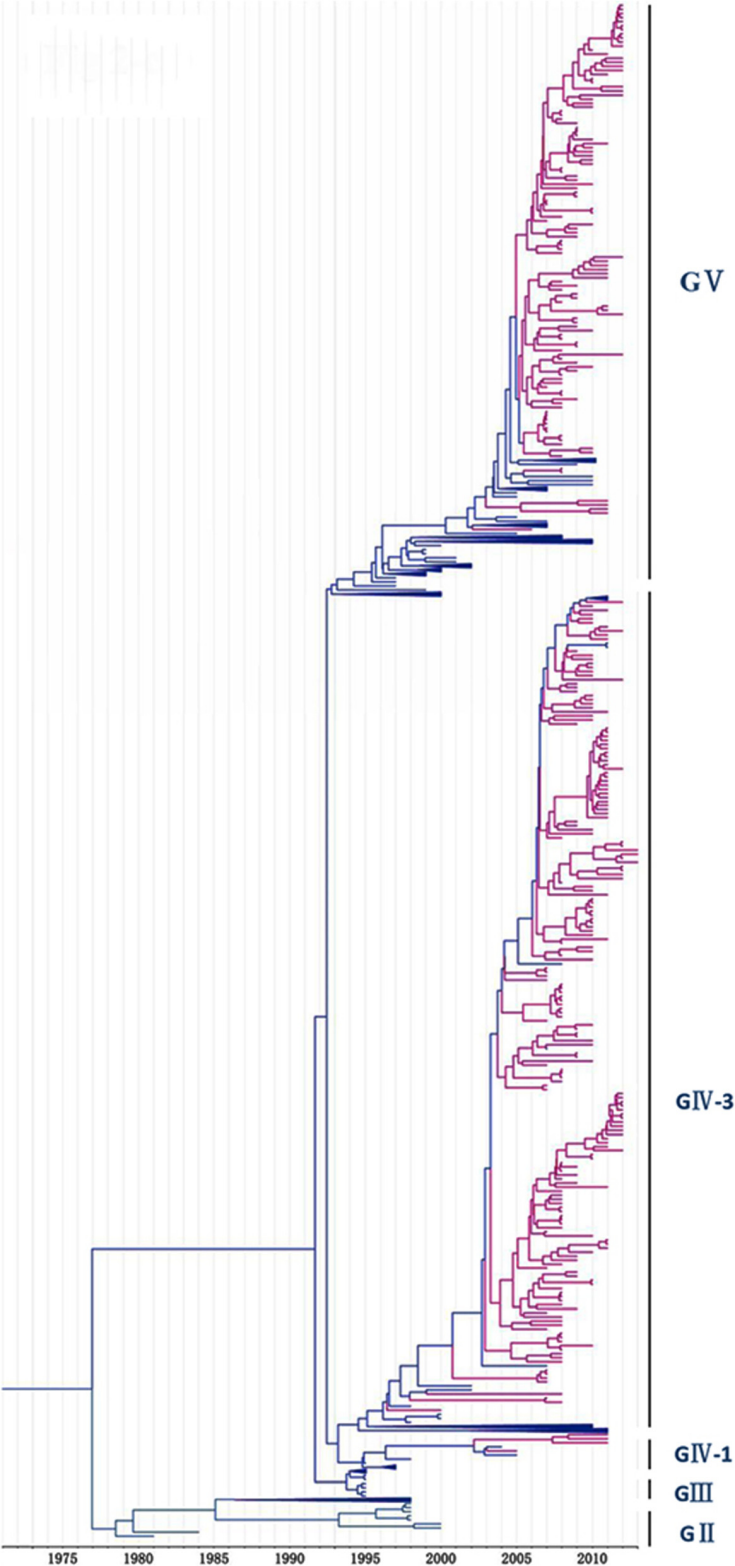
Phylogenetic analysis of CV-A16 from China. The scale is in units of evolutionary time in years. Phylogenetic tree of 81 China strains from 2000 to 2013. The branches of sequences from China are highlighted in red

**Fig. 5:**
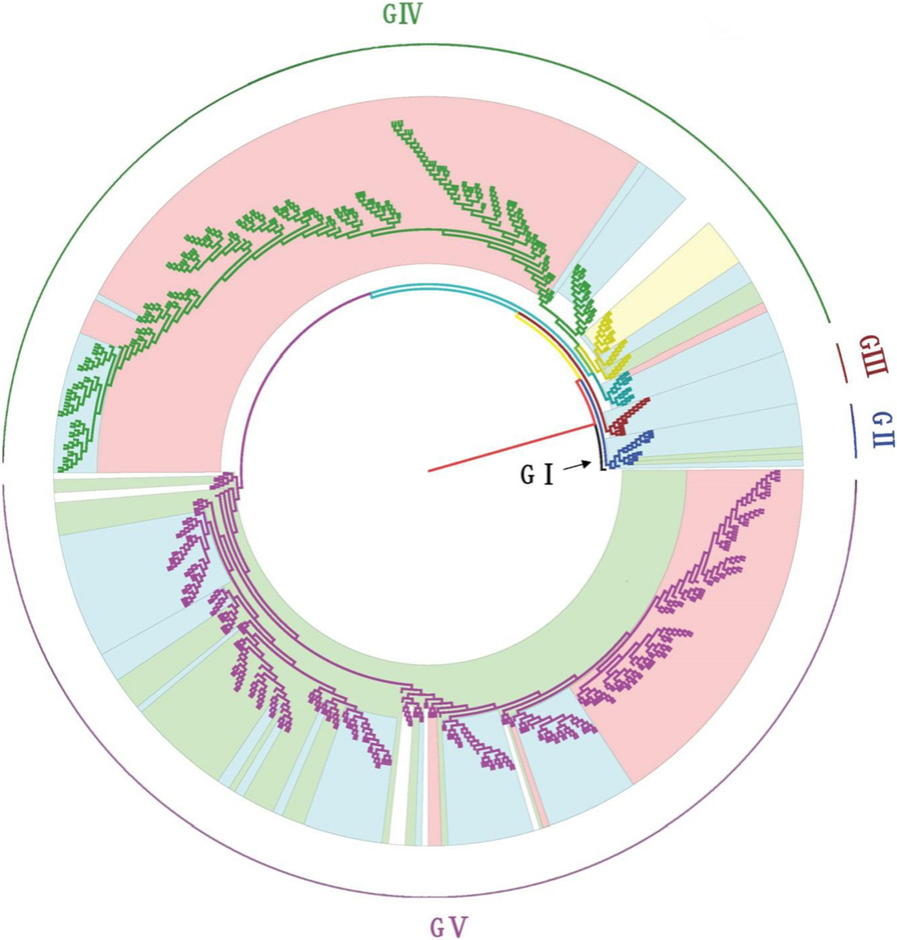
Origin and distribution of CV-A16 from Japan, Malaysia, and China. Different background colors distinguish countries. The blue color depicts Japan (*n* = 255), and green and pink colors display Malaysia (*n* = 82) and China (*n* = 315), respectively

It was shown that the CV-A16 that co-circulated in China since 2007 were clustered strains of genogroups GIV-3 and GV, except for four strains of genogroup GIV (Wuhan0232/HuB/CHN/2011, Wuhan0289/HuB/CHN/2011, Wuhan0158/HuB/CHN/2011, and Wuhan0136/HuB/CHN/2011) and two strains of genogroup GI (FY18 and CA16v-2010221). Among the 315 strains isolated in China, 195 and 118 strains could be assigned to genogroups GIV and GV, respectively, by phylogenetic analysis (Fig. 4). The first strain of sub-genogroup GIV-3 (shzh00-1) documented in mainland China was detected in 2000 in Guangzhou province [25]. The GIV-3 epidemic of 1998–2000 in China originated from a Japanese genogroup (Fig. 5). Genogroup GV is another dominant genogroup, which has been co-circulating with GIV-3 since 2006 that closely resembles the Japanese strains. Figure 1 shows that the CV-A16 serotypes circulating recently in China have the same ancestor, suggesting that the epidemic strains are native and have not invaded the region since 2007. The CV-A16 epidemic strain isolated in the Jilin province, China, can be classified in the GIV-3 sub-genogroup.

### Genetic diversity analysis with Bayesian skyline plot

**Fig. 6:**
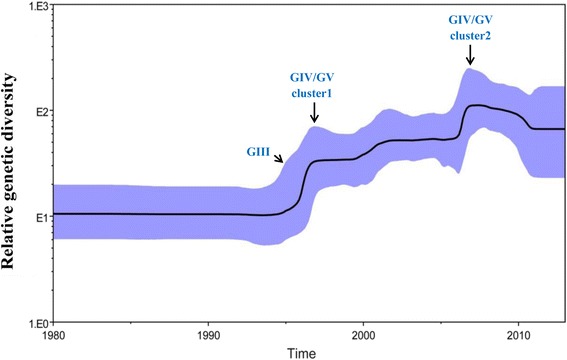
The genetic diversity dynamics of the *CV-A16 VP1* gene estimated by a Bayesian skyline plot through time. A dashed line indicates the mean, whereas shaded areas show the upper and lower 95 % HPD values. The horizontal axis is in the unit year, and the vertical axis is the Net (the product of the effective population size and the generation length in radiocarbon years). The plot for *CV-A16 VP1* shows a continuous increase since the mid-1990s, with sharp increases in genetic diversity in 1997 and 2007. Very low genetic diversity existed in the early 1990s and in 2010

To reconstruct the evolutionary epidemiology of CV-A16, we used Bayesian skyline plot analysis by measuring the dynamics of *CV-A16 VP1* genetic diversity over time (Fig. 6). It can be observed from the evolutionary epidemiology that *CV-A16 VP1* genetic diversity continuously increased since the mid-1990s, indicating evolution from genogroup GII to GIII. This trend coincided with the major HFMD outbreak, mostly caused by the CV-A16 pathogen, in Japan in 1995. A sharp but transient increase in relative genetic diversity was observed for the *CV-A16 VP1* gene between 1996 and 1997. Since 1997, several large epidemics of HFMD have been reported in the Asia–Pacific region, especially in Southeast Asia. Outbreaks with multiple cases have occurred in Taiwan, Malaysia, and Singapore [11, 25, 26]. These revealed the evolution of CV-A16 genogroup GIII to genogroups GIV and GV around 1996–1997. From 1999 to 2001, the genetic diversity of *CV-A16 VP1* underwent a smooth and steady rise, reflecting the transient but sporadic occurrence of CV-A16 outbreaks in various parts of the world. In late 2000, a recurrence of an outbreak of HFMD occurred in Malaysia, with eight deaths in peninsular Malaysia [25]. From 2006 to 2007, another sharp increase emerged for the genetic diversity of *CV-A16 VP1*, representing the emergence of a new branch of the genogroups GIV and GV. After 2007, the cluster 1 of genogroups GIV-3 and GV were replaced by cluster 2, and transmission of these led to a new HFMD outbreak in China and Malaysia [20, 27]. CV-A16 is reported to have accounted for 90 % (399/417) of all enteroviruses causing HFDM in Malaysia in 2007 [27]. Since 2007, the Bayesian skyline plot of CV-A16 showed that its genetic diversity decreased and was maintained corresponding to the MCC (Fig. 1) no new sub-genogroup emerged. Due to the stability of CV-A16 after 2010 and accumulated population immunity, CVA6 has become the main pathogen of HFMD disease substituting for CV-A16 in recent years [13, 28, 29].

### Codon substitution and evolution rates of *CV-A16 VP1* gene

To understand the evolution of CV-A16, we estimated codon substitution and evolution rates using the Bayesian MCMC method. All three codon positions of the *CV-A16 VP1* gene had different relative substitution rates (Table 2). The mean values of the first, second, and third codon positions were 0.244, 0.018, and 2.67, respectively. Among these codon positions, the relative substitution rate of the third codons was highest. Simultaneously, our analysis showed that the *CV-A16 VP1* gene evolutionary rate was estimated to be 6.656E-3 substitutions per site per year (3.978E-4, 2.456E-3; 95 % HPD), inferred by the models of HKY, approximated to estimates of EV-A71 and much less frequently than the polio virus (1.036 E-2) that was estimated also by the MCMC method previously [18, 30].

**Table 2:** Estimates of the relative substitution rates for the core gene of all three codon positions

Summary statistic	CP1.mu	CP2.mu	CP3.mu
Mean	0.268	0.108	2.624
95 % HPD lower	0.234	0.091	2.592
95 % HPD upper	0.295	0.124	2.656
Effective sample size (ESS)	7502	8586	7621

## Discussion

This study establishes the phylogenetic relationship, genomic diversity, and the evolutionary rate of CV-A16 for the first time using the Bayesian Markov chain method, providing new sights into the relationship of evolutionary history of virus population and disease periodicity. We reconstructed the epidemic history of CV-A16 and found that the CV-A16 virus, prevalent between 1980 and 2013, is a pathogen that originated around mid-twentieth century.

Bayesian derivation and the maximum likelihood method have similar characteristics in that both have excellent statistical characteristics. However, one difference is that the Bayesian method can use posterior probability, which is derived from the Markov chain to optimize criterion [31]. A very accurate posterior probability can be obtained using the Bayesian MCMC method due to the rigorous control criterion of every link. Against this background, we established the phylogenies of the CV-A16 gene based on Bayesian derivation combined with the Markov chain model method. Compared with the phylogenetic analysis reported in the previous study [8] using the Neighbor-joiningmethod, the CV-A16 causing the HFMD outbreak in Yamagata, Japan, in 1995 can be independently classified under genogroup GIII using the Bayesian method (Fig. 1, Table 1). There were swift but sporadic occurrences of HFMD in Japan in various years such as 1984, 1988, and 1991 [32]. However, the nucleotide sequence of CV-A16 were relatively stable in this period, reflecting that the epidemic disease that occurred every several years was determined by the cumulative proportion of unvaccinated children and not by the viral antigen’s evolution [32]. Four years late, in 1995, an HFMD outbreak was reported in Japan, which was different from the past episodes; CV-A16 became the main pathogen that replaced EV71 [32, 33]. This suggested that a new CV-A16 genogroup emerged different from former CV-A16 epidemic strains. From the results of our Bayesian skyline plot (Fig. 6), we can also survey the great change in the genetic diversity of CV-A16 in 1995. Therefore, the MCC we reconstructed on the basis of spatiotemporal divergence and genetic diversity is consistent with the trends of epidemic disease. It is very interesting that genogroup GI disappeared for almost 60 years and then it is detected again in 2010 both in our and previous reports [12]. We do not know the reasons for such large-scale changes for genogroup GI, but they may be associated with the G-10’s weakly pathogenicity which didn’t cause enough attention and lead to a lack of continuity monitor data.

From the Bayesian skyline plots (Fig. 6), we can see that every sharp change of genetic diversity resulted in a large-scale HFMD outbreak. To some extent, the increase of genetic diversity corresponding to this characteristic since mid-1990 was a marker for the emergence of a new CV-A16 genogroup. The data set also indicated agreement between genetic diversity dynamics and emergent genogroups, which reflect the earlier HFMD outbreaks. Since 2007, the genetic diversity of CV-A16 stabilized and slightly decreased, and no novel genogroup emergence was reported [12, 13, 33]. However, there were some HFMD outbreaks caused by CV-A16 in various provinces of China that may be attributable to the cumulative proportion of unvaccinated children and increased detection intensity. Since 2010, genetic diversity dynamics tended to be gentler, CVA6 replaced CVA16 become the second pathogene in Shenzhen and Guangdong, China [34, 35].

## Conclusions

CV-A16 has long been the main pathogen of HFMD, seriously threatening human health [8, 11, 12, 33]. The reports of some deaths caused by CV-A16 infection [2, 36, 37], suggesting that more attention should be paid to the detection and prevention of CV-A16. From the data we obtained, we predicted the dynamic phylogenetic trends, which indicate outbreak trends of CV-A16, and provide theoretical foundations for clinical prevention and treatment of HFMD which caused by a CV-A16. The relatively stable nucleotide sequence will provide a great opportunity to develop a vaccine for this disease. So the development and administration of its vaccine should be accelerated.

## Additional files

Additional file 1:
**Table S1.** Primers, probes and PCR amplification condition in this study. (DOCX 17 kb) Additional file 2:
**Table S2.** List of the 708 complete VP1 sequences of CVA16 strains available from GenBank which were selected to generate the CVA16 MCC. (XLSX 38 kb) Additional file 3: 160 typical CV-A16 VP1 used to reconstruct the population history of CV-A16.(PDF 38 kb) Additional file 4: The exact time and location of 708 sequences between 1951 and 2013.(PDF 36 kb)
