# Supplementary material for: Characterization of VP1 sequence of Coxsackievirus A16 isolates by Bayesian evolutionary method
Source: Virol J. 2016 Jul 28;13:130. doi: 10.1186/s12985-016-0578-3 (PMC4963925; doi:10.1186/s12985-016-0578-3)
Supplement: Additional file 1: — Table S1. Primers, probes and PCR amplification condition in this study. (DOCX 17 kb) [file 12985_2016_578_MOESM1_ESM.docx]

**Table S1**

Primers, probes and PCR amplification condition in this study

|  | Primer | Sequences(5'-3') |  |
| --- | --- | --- | --- |
|  | EV-F | CAAGCACTTCTGTTTCCCCGG | 435bp |
|  | EV-R | ATTGTCACCATAAGCAGCCA |  |
|  | EV71S-F | GTGGCAGATGTGATTGAGAG | 332bp |
|  | EV71S-R | GTTATGTCTATGTCCCAGTT |  |
|  | CVA16S-F | TTGCAGACATGATTGACCAG | 211bp |
|  | CVA16S-R | GAGTGATGGTTCAACACACA |  |
|  | CVA16VP1-F | ATTGGTGCTCCCACTACAGC | 1110bp |
|  | CVA16VP1-R | GCTGTCCTCCCACACAAGAT |  |
