# Supplementary material for: Characterization of VP1 sequence of Coxsackievirus A16 isolates by Bayesian evolutionary method
Source: Virol J. 2016 Jul 28;13:130. doi: 10.1186/s12985-016-0578-3 (PMC4963925; doi:10.1186/s12985-016-0578-3)

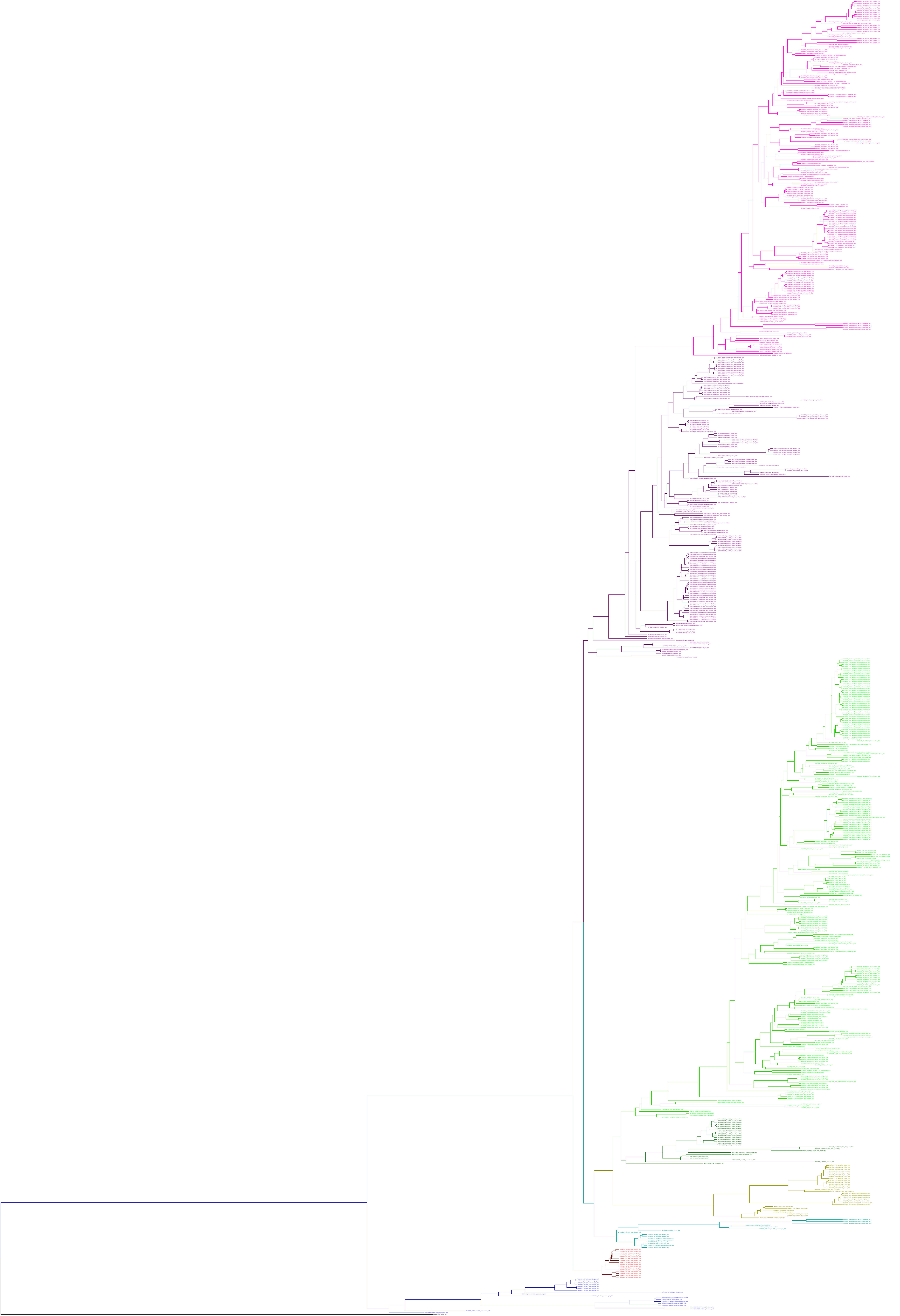

Supplement: Additional file 3: — 160 typical CV-A16 VP1 used to reconstruct the population history of CV-A16.(PDF 38 kb) [file 12985_2016_578_MOESM3_ESM.pdf]
